# The complete mitochondrial genome of a fouling mussel, *Xenostrobus atratu*s (Mollusca: Mytilidae), and its phylogenetic implication

**DOI:** 10.1080/23802359.2023.2179354

**Published:** 2023-02-24

**Authors:** Houmei Li, Chenghua Li, Peizhen Ma, Haiyan Wang, Zhen Zhang

**Affiliations:** a School of Marine Sciences, Ningbo University, Ningbo, P. R. China; b Laboratory of Marine Organism Taxonomy and Phylogeny, Qingdao Key Laboratory of Marine Biodiversity and Conservation, Institute of Oceanology, Chinese Academy of Sciences, Qingdao, P. R. China; c University of Chinese Academy of Sciences, Beijing, P. R. China

**Keywords:** *Xenostrobus atratus*, mitochondrial genome, phylogenetic analysis

## Abstract

In this study, we report the female-lineage mitochondrial genome of *Xenostrobus atratus* for the first time. The circular mitochondrial genome is 14,806 bp in length and contains 12 protein-coding genes, 22 transfer RNA genes, and two ribosomal RNA genes. All genes are encoded on the heavy strand. The genome composition is A + T biased (66.6%), with 25.2% A, 41.4% T, 21.7% G and 11.7% C. A Bayesian inference (BI) phylogenetic tree was constructed based on the mitochondrial genomes of *X. atratus* and 46 other Mytilidae species. Our results demonstrate that *X. atratus* and *Limnoperna fortune*i have distinct lineages, opposing synonymizing *Xenostrobus* within *Limnoperna*. According to this study, the validity of the subfamily Limnoperninae and genus *Xenostrobus* is strongly supported. However, there is still an urgent need for more mitochondrial data to decide to which subfamily *X. atratus* belongs.

## Introduction

*Xenostrobus* species occupys different habitats, such as tropical mangroves, rocky substrates upstream and downstream of estuaries, and rocky intertidal zone (Colgan and da Costa 2013). There are eight extant species of *Xenostrobus* (Colgan et al. 2020), and only one, *Xenostrobus atratus*, is reported for China (Bernard 1993; Wang 1997). Due to the similarity of morphological characteristics with an invasive freshwater mussel, *Limnoperna fortunei* (Beu 2006), *X. atratus* used to be named *Limnoperna atrata*. The relationship between the two genera is still in debate. For example, Beu (2006) placed the species within the *Limnoperna* genus, however, Colgan and da Costa (2013)-based on differences in BEAST-estimated age and *cox1* amino acid sequences-objected to the suggestion that *Xenostrobus* and *Limnoperna* were congeneric. Therefore, convincing evidence is needed to consider the relationships between *Xenostrobus* and *Limnoperna*. In addition, the fouling property that affects the normal growth and development of mangroves (He, 2002) draws considerable attention to *X. atratus*. Since characteristics of mitochondrial genomes are efficient and powerful in phylogenetic studies in Mytilidae (Lee et al. 2019; Zhang et al. 2019), the mitochondrial genome of *X. atratus* was sequenced for the first time in this study. An interesting feature of Mytilidae mtDNA is Doubly Uniparental Inheritance (DUI), which has two types of mitochondrial DNA [female-lineage type (F-type) and male-lineage type (M-type)] and M-type mitogenome is only present in the gonads of male individuals. In mytlids, the F-type and M-type mitogenomes appear to exhibit an estimated 10% difference in base sequences (Fisher and Skibinski, 1990). DUI species were found not only in marine mussels but also in freshwater mussels (Soroka, 2020). There are more than 100 species of bivalve mollusks known to have DUI (Zouros, 2020).

## Materials and methods

### Mussel materials and DNA extraction

The specimen was collected from Qingdao, Shandong Province, China (119°58′12″N, 35°52′48″E) on 4 February 2022 and was stored in the Marine Biological Museum, Chinese Academy of Sciences (http://www.qdio.cas.cn/, Yongqiang Wang, bmxia@qdio.ac.cn, under the voucher number MBM287353). Genomic DNA was extracted from the adductor muscle by CTAB method (Doyle and Doyle 1987).

### Genome sequencing, assembly and annotation

The *X. atratus* DNA library was sequenced by Personalbio Biotechnology Co., Ltd (Shanghai, China) using an Illumina NovaSeq 6000 with an average insert size of 400 bp, which was constructed using TruSeq^TM^ Nano DNA Sample Pre Kit. Approximately 3.98 GB of raw data for *X. atratus* were generated with 150 bp paired-end reads. Adapters were removed by Adapter Removal v.2 (Schubert et al. 2016). Clean data was assembled de novo using A5-miseq v20150522 (Coil et al. 2015) and SPAdesv3.9.0 (Bankevich et al. 2012). We determined the location of genes using the MITOS web server (http://mitos2.bioinf.uni-leipzig.de/index.py). The annotated sequence has been submitted to GenBank with Accession no. OM001008 (F type).

### Phylogenetic analysis

A Bayesian inference (BI) phylogenetic tree was conducted using sequence data from the concatenated sets of 12 PCGs and 2 rRNAs of the *X. atratus* mitogenome and 46 Mytilidae species previously published, with *Crassostrea gigas* (Ostreidae) and *Anadara sativa* (Arcidae) as outgroups. The best substitution model for nucleotide sequences selected by Modelfinder (Kalyaanamoorthy et al. 2017) was GTR + F + G. BI tree was established using software MrBayes v.3.2.6 (Ronquist et al. 2012)^,^ with 2,000,000 generations and discarding the first 25% as burnin.

## Results

The mitochondrial genome of *X. atratus* is a closed-circular DNA molecule with a length of 14,806 bp, containing 12 protein-coding genes (PCGs), including 7 subunits of NADH dehydrogenase (*nad1-6* and *nad4l*), 3 subunits of cytochrome c oxidase (*cox1-3*), one subunits of ATPase (*atp6*), cytochrome b (*cytb*), 22 transfer RNA genes (tRNAs) and 2 ribosomal RNA genes (*12S* rRNA and *16S* rRNA). The total length of the non-coding regions is 449 bp, accounting for 3.03% of the mitochondrial genome. No *atp8* gene was found by comparison with closely related species. Song et al. (2009) hypothesized that the absence of gene *atp8* in some species resulted from adaptation to environmental conditions, but, this coding gene has been detected in many fresh-water and marine bivalves. This might be explained by the inherent structural properties (such as short length) and extreme variability of *atp8* among bivalves that hider the proper detection and annotation of this gene (Breton et al. 2010; Gaitán-Espitia et al. 2016).

**Figure 1.**
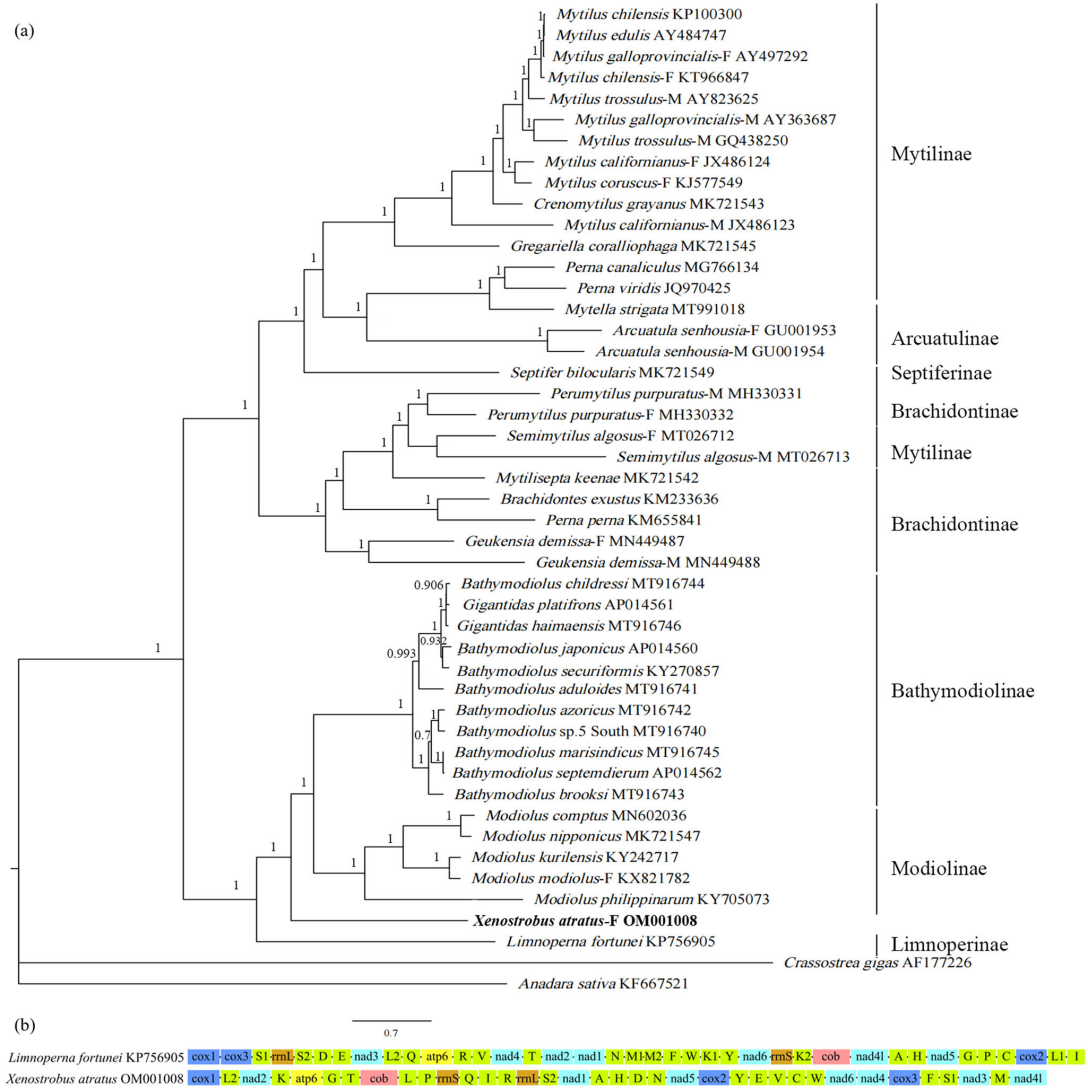
(a) The Bayesian inference phylogenetic tree for *Xenostrobus atratus* and other Mytilidae species, with numbers next to the node are support values; (b) Mitochondrial genome arrangement of *Xenostrobus atratus* and *Limnoperna fortunei*. The -F represents female-lineage mitochondrial genome and -M represents male-lineage mitochondrial genome.

The phylogenetic tree divides the 46 Mytilidae species into 2 clades (Figure 1(a)), one of which, clusters *X. atratus* with Bathymodiolinae, Modiolinae, and Limnoperninae.

## Dicussion

The validity of the subfamily Limnoperninae has been doubted and the genus *Limnoperna* was sometimes placed under Arcuatulinae (Huber 2010). In this study, *L. fortunei* clusters with *X. atratus*, Bathymodiolinae and Modiolinae, constituting one of the two main clades in Mytilidae, and not with Arcuatulinae species. Consequently, it is more likely that Limnoperninae is valid. Lee et al. (2019) proposed that Limnoperninae is sister to (Bathymodiolinae + Modiolinae) based on a mitochondrial genome phylogeny, but the newly sequenced species, *X. atratus*, takes its position and clusters with (Bathymodiolinae + Modiolinae) in this study, and they are then sister to Limnoperninae (posterior probability = 1). In particular, *X. atratus* and *L. fortunei* are closely related but have distinct lineages with long phylogenetic branch lengths, longer than most of the other closely related species within the Bathymodiolinae and Modiolinae, opposing the synonym of the two genera raised by Beu (2006). Additionally, since gene orders among low-level taxonomic species are highly conserved and have been proven to be an effective phylogenetic tool in Mollusca (Boore and Brown, 1998; Ghiselli et al. 2021), the very large difference in the PCG order and tRNA arrangement between *X. atratus* and *L. fortunei* provides further evidence for supporting the validity of genus *Xenostrobus* (Figure 1(b)). Nonetheless, it’s still too early to decide which subfamily *X. atratus* belongs to based on current studies. Additional molecular data are needed to solve this controversy and deeper relationships among Mytilidae species.

## Data Availability

The genome sequence data that support the findings of this study are openly available in GenBank of NCBI at (https://www.ncbi.nlm.nih.gov/) under accession no.OM001008. The associated BioProject, SRA and Bio-Sample numbers are PRJNA808856, SRR18136193, and SAMN26116627 respectively.
